# Fish Consumption Caveat: Advisories May Not Help with Long-Lived Contaminants

**DOI:** 10.1289/ehp.122-A57

**Published:** 2014-02-01

**Authors:** Kellyn S. Betts

**Affiliations:** Kellyn S. Betts writes about environmental contaminants, hazards, and technology for solving environmental problems for publications including *EHP* and *Environmental Science & Technology*.

Worldwide, people’s diets are their main source of exposure to most persistent organic pollutants (POPs).^1^^,^^2^ Fish is a major contributor to POP intake and also the primary dietary source for methylmercury.^3^ A new statistical modeling tool discussed in *EHP* suggests that fish consumption advisories are unlikely to effectively reduce prenatal exposures to polychlorinated biphenyls (PCBs) and other POPs with long elimination half-lives.^4^

The model, dubbed CoZMoMAN, was developed by a team of computer modeling researchers. CoZMoMAN is actually a combination of two mechanistic models, one devoted to environmental transport and distribution of pollutants, and one that models bioaccumulation in the human food chain. The combined models can estimate a chemical’s trajectory through the physical environment and the food chain into the human body in “one grand calculation,” says coauthor Frank Wania, a professor in the University of Toronto Scarborough’s Department of Physical and Environmental Sciences.

The researchers’ goal was to study how compliance with fish advisories, levels of environmental contamination, and behavior of chemicals within the human body influenced exposures in children from conception through age 9 years. They focused on PCB-153, which has a half-life in humans of about 15 years.^5^

**Figure:**
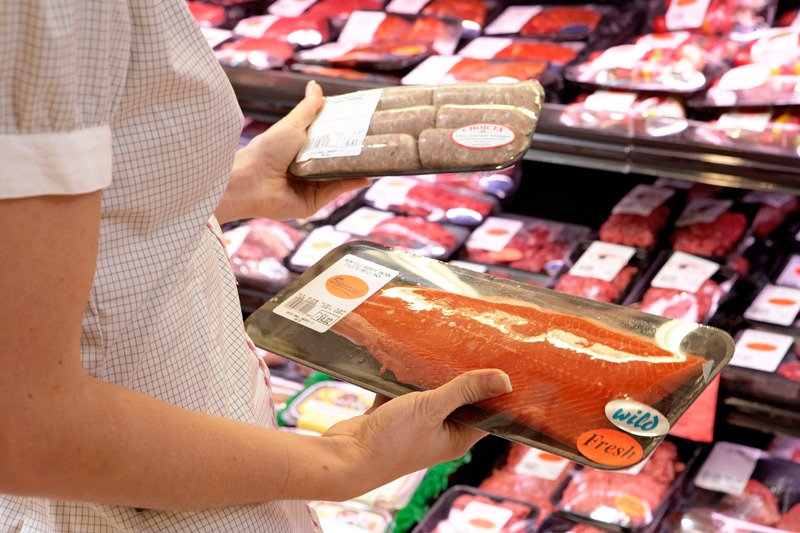
Fish advisories are most helpful when they include fact-based recommendations for healthy alternatives. © Noel Hendrickson/Getty

For two generations of hypothetical children the model estimated ancestral exposures using historical dietary and emissions data to predict lifetime POP exposures. The model assumed grandmothers gave birth at age 30 and breastfed their daughters for 6 months. The daughters, in turn, gave birth at 30 and breastfed their own children for 6 months. Daughters complied with fish consumption advisories for either 3 months or 5 years prior to conceiving their children and replaced either half or all of their default 76-g/day serving of fish with either fruits and vegetables (typically not a source of PCB exposure) or with beef. The hypothetical 30-year-old daughters consumed most of their protein in the form of dairy, which can also be contaminated with POPs. Children were assumed to follow their mothers’ diet after weaning.

The model estimated that children’s PCB-153 uptake to 9 years of age could be reduced only if contaminants had time to clear from the body during their mothers’ period of compliance with the fish advisory—not a realistic proposition with the long-lived PCB-153. Assuming PCB-153 emissions remained steady, the only scenarios that produced a significant impact on children’s exposures required mothers to eliminate fish from their diets for 5 years before their children were conceived. The model predicted that substituting produce for fish would reduce prenatal and breastfeeding exposures by 37% each and subsequent childhood exposures by 23%.^4^

In contrast to the steady-state assumption, emissions of legacy PCBs have been steadily declining since the mid-1970s. In alternative scenarios modeling this decline, the effect of dietary switches was dampened in children born after emissions peaked.^4^ However, even very small decreases in exposure to POPs could have a beneficial impact during critical periods in fetal development, says Joanna Burger, a behavioral ecologist at Rutgers University.

Emily Oken, an associate professor at Harvard Medical School’s Department of Population Medicine, says the model’s most interesting data relate to the potential for chemical uptake trade-offs. For example, under the steady-state scenario, the model predicted that replacing all fish with beef for 5 years would result in slight declines in PCB-153 uptake but could expose people to other contaminants with different potentially harmful properties, including β-endosulfan, β-hexachlorocyclohexane, and hexachlorobenzene.^4^

This underscores the value of including specific recommendations for healthy alternatives to fish in advisories. Health practitioners generally advise expectant mothers not to avoid all seafood, since fish is an excellent source of the omega-3 polyunsaturated fatty acids essential for healthy neurological development.

A preliminary analysis suggested that compliance was more likely to have an appreciable impact on exposure to methylmercury due to its relatively short 40- to 50-day half-life in humans. However, the authors caution that the model must be refined to account for mercury bioaccumulation.^4^

A potential limitation of the study is the model’s reliance on consumption data based on Swedish diets. Consumption patterns vary dramatically both regionally and ethnically even within the United States, Burger points out. Wania says his group is currently expanding the model to include information about contaminants associated with other animal-based food. They’re currently working on foods preferred by Arctic natives, such as beluga whale, caribou, and moose, but they may eventually research the impacts associated with more commonly eaten meats such as poultry and pork.

The model has potential for helping governments make good decisions about where to focus public health efforts to have the largest beneficial impact, Oken concludes. “We don’t want to be giving advice that’s actually not helping people,” she says.
